# Three-Dimensional Modeling of Tea-Shoots Using Images and Models

**DOI:** 10.3390/s110403803

**Published:** 2011-03-29

**Authors:** Jian Wang, Xianyin Zeng, Jianbing Liu

**Affiliations:** 1 College of Biology and Science, Sichuan Agricultural University, Ya’an 625014, Sichuan, China; E-Mail: xyzeng@sicau.edu.cn; 2 College of Electronic Engineering, University of Electronic Science and Technology of China, Chengdu 610054, Sichuan, China; E-Mail: C200512@sina.com

**Keywords:** tea shoots, three-dimensional modeling, calculation model, image segmentation, edge detection

## Abstract

In this paper, a method for three-dimensional modeling of tea-shoots with images and calculation models is introduced. The process is as follows: the tea shoots are photographed with a camera, color space conversion is conducted, using an improved algorithm that is based on color and regional growth to divide the tea shoots in the images, and the edges of the tea shoots extracted with the help of edge detection; after that, using the divided tea-shoot images, the three-dimensional coordinates of the tea shoots are worked out and the feature parameters extracted, matching and calculation conducted according to the model database, and finally the three-dimensional modeling of tea-shoots is completed. According to the experimental results, this method can avoid a lot of calculations and has better visual effects and, moreover, performs better in recovering the three-dimensional information of the tea shoots, thereby providing a new method for monitoring the growth of and non-destructive testing of tea shoots.

## 1. Introduction

Three-dimensional modeling of plant morphology is currently an important topic in the field of agriculture and machine vision, as it can exactly reflect the morphology of plants and analyze the qualitative features and physiological and ecological processes [1–3]. In recent years, more and more attention has been paid to the exact measurement and three-dimensional modeling of plants’ morphological parameters using images and videos. Previously, such research mainly took trees as the research objects and then gradually switched to the crops like corn, wheat, *etc*. [4–6]. With images and the L system, Shlyakhter, *et al.*, reconstructed tree configuration by constructing a virtual shell [7]. Reche, *et al.*, conducted body modeling of trees by the transparency of images [8]. By measuring and analyzing the botanical characteristics (e.g., the leaf area index), Rakocevic *et al.*, measured the three-dimensional feature points of white clover using a three-dimensional digitizer [9]. According to the solid visual principle, Telin, *et al.*, studied the three-dimensional modeling seedings and, using the system, measured parameters like leaf area [10]. Hammel, *et al.*, established a model for complex plant leaves using the limb frameworks, while Hammel, *et al.*, studied the image-based surface modeling of plant organs [11–13] and Thies *et al*. studied the application of laser scanners in 3D reconstruction [14].

The traditional organ modeling methods, such as the L system-based leaf modeling, are mainly based on geometric graphics [15]. The controllability of the model is good, but the visual effect is not satisfactory. Thus, to get a better effect, the operator must perform highly complex graphical operations. Image-based organ modeling gives good visual effects but poor growth controllability. At present, most of the research focuses on three-dimensional modeling and simulation of the plant configuration using solid visual systems and three-dimensional digitizers [16]. However, during the modeling of the solid visual system, accurate interaction is required to guarantee the preciseness of matching and the laser scanning has very rigorous color reproduction requirements, while the cost of the modeling using a three-dimensional visualization instrument or laser scanning are too high. Moreover, some problems, such as low measurement accuracy, insufficient fidelity and the need for a rigorous image acquisition environment exist in the traditional methods.

In recent years, modeling technology combined with images has enjoyed a rapid development. Due to its help, we can acquire better visual effect with less computation, and the digital devices are relatively cheaper. Ya’an is one of the ancient birthplaces of tea in China and a huge area of tea is cultivated in Menting Mountain. The growth status and maturity have an important impact on the tea picking and quality of the finished products, and thus duly knowing the growth status of tea shoots is of positive significance to the standardized large scale production of tea [17]. So far, there haven’t been any studies on the precise measurement and three-dimensional modeling of tea leaves. In this paper, based on the previous studies, a study on the three-dimensional shape of tea shoots is carried out, and a method for three-dimensional modeling of tea shoots with images and calculation models is introduced.

## 2. Overview of the Tea-Shoot Modeling System

**Figure 1.**
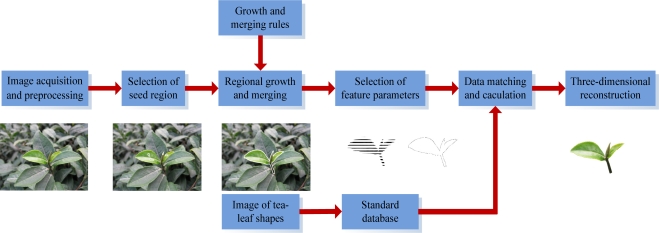
Overview of the tea-shoot modeling system.

In this paper, a three-dimensional modeling system for tea shoots with images and models is introduced, as shown in Figure 1. The process is: the tea shoots are photographed with a camera, the denoising and color space conversion from RGB to HSI is carried out, and then an improved algorithm that is based on color and regional growth is used to divide the tea shoots in the images and extract the tea shoot edges with the help of edge detection; next the three-dimensional coordinates of branches and leaves are worked out with the acquired images of tea shoots, and the feature parameters of tea shoots’ contours: axis, leaf width, leaf length and leaf inclination angle, branch length and diameter are extracted. At the same time, the collection of digital images regarding tea shoots and young leaves is acquired, their feature parameters extracted, and then calculation models related to the status of tea shoots and young leaves are constructed and the model database of tea leaves is established by analyzing their features. Then, by matching and computing the acquired tea shoot parameters with the existing model database, the leaf configuration and framework are worked out by matching the leaf images in the database, and then VC++ plus OpenGL are used to complete the three-dimensional modeling of tea shoots [18,19]. This method avoids the complicated calculations required for pure mathematical model modeling and also avoids the huge amount of images needed in pure image-based modeling.

## 3. Segmentation of Tea Shoots

Accurate segmentation of tea shoots is the basis for the follow-up feature extraction and three-dimensional modeling. In this study, according to the color space characteristics of tea-tree images and similarity & dissimilarity relations of regional features between the tea shoots and mature leaves, a new algorithm based on regional growth was employed to conduct the regional growth and merging by taking the color distance and edge distance into account. The regional growth merging method can effectively fuse the color information and space information and, at the same time, integrate the local and general information [20]. Thus, it can merge and divide the non-neighboring regions with similar colors in space and, moreover, integrate the local and general information with the color and region features, thereby carrying out the segmentation of tea shoots in a better manner.

### 3.1. Selection of the Seed Region

To reduce the impact of brightness changes in images on the segmentation effect, we converted the images from their RGB color space to the HIS color space and, at the same time, selected the H and S parameters to select the seed region. In the selection of seed region, some pixels in the image will be used to represent the seeds, and thus a small region can be regarded as a seed region when the pixels have same color in color quantization and are connected with the surrounded pixels to reach certain area. Then, if having the same characteristics with the seed region, the neighboring regions that are connected with the seed region will be added to the seed region.

### 3.2. Regional Growth and Merging

Upon the selection of seed region, the regional growth was determined according to the color similarity of tea shoots. Considering that the block characteristics of the same target region and the neighboring regions are almost the same to each other, we scanned and merged many sub-regions in the images. For the neighboring and similarity among regions, the regional growth and merging was conducted according to the established rules of reasonable merging and stopping merging, thus achieving a fast and accurate segmentation of the tea-shoot images.

#### 3.2.1. The Distance Definition of Color

In the tea-leaf images, if two regions are similar in color and neighboring in space without an obvious borderline between them, they can be regarded as two connected regions. That is, the maximum value of the relative color distance between a region and its neighboring regions should be less than the defined threshold (threshold is taken as 12 in the experiments) [21]. The distance definition of color can be computed with the mean hue component value, which is defined as below:

(1) $$D_{c}=\frac{r_{i}\cdot r_{j}}{r_{i}+r_{j}}\left\|{\bar{\mu }}_{i}-{\bar{\mu }}_{j}\right\|$$

where *r*_i_ and *r*_j_ represent the number of pixels in the *i* and *j* regions, while *μ̄_i_* and *μ̄_j_* represent the mean color values of the both regions, and ‖ ‖ represents the Euclidean distance. The product of *r*_i_ and *r*_j_ makes that the color distance of the region with less pixels is smaller than that of the other regions, which, if the mean color value is the same, will give priority to the merging of small regions, thus making the segmentation results fit human beings’ visual features better. For the initial partition, the hierarchical region merging algorithm was introduced to carry out the final segmentation [22]. That is, forming a new region and then adjusting the neighboring relation and distance between the new region and other regions correspondingly.

#### 3.2.2. Merging of Regions

During the selection of seed region, several seed regions might be selected out due to the fact that there might be similar sub-regions in the mature leaves, resulting in errors or over-segmentation [23]. To overcome this problem, we introduced the edge distance to distinguish the tea shoots and mature leaves and laid some restrictions on the region merging. The definition of the edge distance ensures that two neighboring regions with different colors can be merged when the changes in color between them is smooth. The edge distance is defined as below:

(2) $$D_{e}=\frac{1}{P_{\mathrm{ij}}}\sum_{m,n}\left\|x_{m}-x_{n}\right\|$$

where *P_ij_* represents the number of pixels along the edge, while *x*_m_ and *x*_n_ represents the color value of the *m* Point and *n* point on the both sides of the edge, and ‖ ‖ represents the Euclidean distance.

## 4. Description of the Morphological Characteristics of Tea Leaves

The upright tea leaf is inserted on the stem with long and hard veins. Thus, to reproduce a realistic tea life in the computer, we need to construct a mathematical model describing the space curves of veins, leaf shapes and characteristics.

On the same branch, the top part is called the terminal bud, followed by tender leaves and young leaves. Generally, the new leaves on the upper part are relatively straight and, with the increase of leaf age, the leaves gradually turn flat and elliptic from top to bottom. With serrated leaf margins and netlike leaf veins, the tea leaves have significant main veins connected with many side veins on the both sides, and the side veins are connected with several sub-veins. The angle between the main and side vein is about 45°–80°. The tea leaves are curved, and there is a certain small-angle folding upward along the main vein.

The status of a tea leaf is significantly different in different stages of shape formation. According to the characteristics of changes in leaf shape, the formation of a tea leaf can be divided into three stages:

1. Bud period: from the beginning of leaf stretching to outspread of the leaf tip and, during this period, the leaf is curved and horn-shaped.
2. Leaf expansion period: the angle between stem and leaf is gradually turning greater, the leaf continues to stretch and spread gradually, and saw-teeth appear along the leaf margin.
3. Leaf formation stage: the elongation of tea leaf will basically stop in this stage, which will last until the full expansion of the leaf.

The model parameters that are used to describe tea shoots have clear biological meanings, including the leaf length, maximum leaf width, space direction of veins, leaf unfolding rate, the maximum vertical difference in height, and leaf obliquity. With these parameters plus the data model and tea-leaf images, the three-dimensional shape of tea shoots can be drawn.

### 4.1. The Vein Curve Model

**Figure 2.**
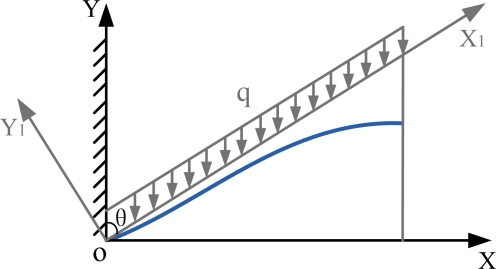
Sketch map of cantilever beam.

From the mechanical point of view, the tea leaf vein can be simplified into a cantilever beam. Supposing that the vein is a cylindrical cantilever beam and it is bent due to gravity, the vein cantilever beam can be indicated in Figure 2. If the mean load on tea leaves is q, the initial angle is θ that is the angle between stem and leaf, E is the elastic modulus and I is the moment of inertia, then the curve equation can be represented as below.

(3) $$E\cdot I\cdot \frac{d^{2}v}{{\mathrm{dt}}^{2}}=-M$$

and $\frac{\mathrm{dv}}{\mathrm{dt}}(0)=\theta$, *v* (0) = 0.

After twice integration, the following equation is acquired:

(4) $$\begin{array}{l} 24E\cdot I(x\;\text{cos}(\theta )+y\;\text{sin}(\theta ))\\ =q\;\text{sin}(\theta )\left[{\left(x\;\text{sin}(\theta )-y\;\text{cos}(\theta )\right)}^{4}-4L{\left(\left(x\;\text{sin}(\theta )-y\;\text{cos}(\theta \right)\right)}^{3}+6L^{2}{\left(x\;\text{sin}(\theta )-y\;\text{cos}(\theta )\right)}^{2}\right] \end{array}$$

Making

(5) $$y_{1}=x\;\text{cos}(\theta )+y\;\text{sin}(\theta )\;\;\;\;\;\;\;\;\;\;\;\;\;\;,\;\;\;\;\;\;\;\;\;x_{1}=x\;\text{sin}(\theta )-y\;\text{cos}(\theta )$$

Then the equation above can be simplified as below:

(6) $$24E\cdot I\cdot y_{1}=q\;\text{sin}(\theta )\left(x_{1}^{4}-4Lx_{1}^{3}+6L^{2}x_{1}^{2}\right)$$

*x*_1_, *y*_1_ are the coordinates in the *X*_1_*OY*_1_ reference frame of Figure 2, and thus the equations are acquired as below:

(7) $$\left\{ \begin{array}{l} x=y_{1}\;\text{cos}(\theta )+x_{1}\;\text{sin}(\theta )\\ y=y_{1}\;\text{sin}(\theta )-x_{1}\;\text{cos}(\theta ) \end{array}\right.$$

In these equations, *L* is the length of vein curve, *θ* is the angle between stem and leaf, *v* is the deflection of the tea leaf vein curve, that is, the distance from the initial vein status. In this system, *E* is a constant number, *I* and *q* are undetermined coefficients. By changing the values of L and θ, we can acquire different curve shapes. The cantilever model can perfectly describe the tea-leaf vein curve with less model parameters but clear biological meanings.

### 4.2. Model of the Tea Leaf Morphology

Based on the results of the previous studies, we conducted a lot of analysis on the test data and put forward a knowledge model of morphological tea-leaf structure [24].

#### 4.2.1. The Relational Model between Leaf Length (LL) and Leaf Order (N)

(8) $$\mathrm{LL}(N)=L_{M}•e^{L_{a}{\left(\frac{N}{N_{M}}-1\right)}^{2}}$$

In this formula, *L_M_* is the maximum length of single leaf, *N_M_* is the leaf order of the branch with the longest leaf (supposed as the variety parameter); La is the model parameter.

#### 4.2.2. The Relational Model of Tea-Leaf Shapes

In the stretching direction, the leaf shape varies along with the change in leaf width, and the function between leaf width (*lw*) and leaf length can be represented as below:

(9) $$\frac{\mathrm{lw}}{\mathrm{LW}}=a•{\left(\frac{\mathrm{ll}}{\mathrm{LL}}\right)}^{2}+b•\frac{\mathrm{ll}}{\mathrm{LL}}+c$$

where LL represents the leaf length, and LW is the maximum leaf width; *lw* is the leaf width when the leaf length is *ll*, and a, b and c are the model parameters.

#### 4.2.3. The Relational Model of Tea-Leaf Area

Leaf area is proportional to the product of leaf length and leaf width:

(10) LA=k×LW×LL

As a correction factor, *k* is associated with the leaf shape and varies along with the tea varieties and leaf order, and it’s usually in the range of 0.67 to 0.8.

## 5. Experimental Results and Discussion

### 5.1. Image Acquisition

**Figure 3.**
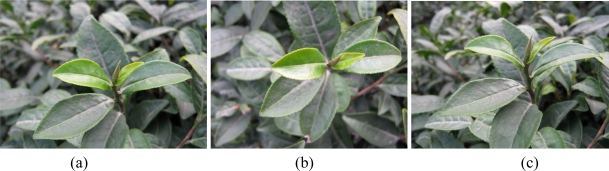
Tea images of different viewing angles [90° (a); 45° (b) and 0° (c)].

In the experiment, the tea shoots were photographed from three angles (between the horizontal line and camera lens): 0°, 45°, and 90°, and then the acquired images were analyzed and divided separately. As shown in Figure 3, at the 0° angle, the contour of the tea shoots can be viewed clearly, and the shapes can be identified easily; however, since it is just the side of tea shoot, we cannot see the complete the tea-shoot shape, and the acquisition of tea-shoot images as well as the accuracy of tea-shoot segmentation are affected. Nevertheless, the two angles (45° and 90°) can perfectly maintain the integrity of the tea-shoot shapes, thus being conducive to the proper tea-shoot segmentation.

According to the experimental results, the images taken at the angle of 90° are better at maintaining the contour of color gamut but incompetent in distinguishing the tea shoots and young leaves. In contrast, the images taken at the angle of 45° are good at distinguishing shoots and mature leaves and, moreover, are more conducive to the proper segmentation and achievement of complete and handsome shapes.

**Figure 4.**
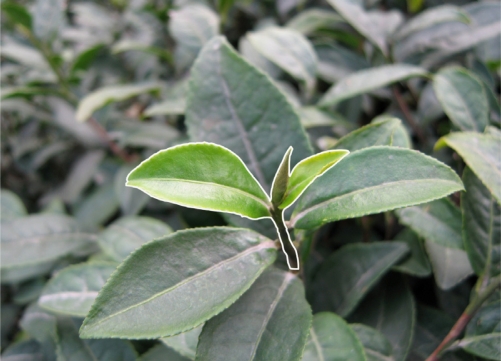
Segmentation of tea-shoot.

In this experiment, a CANON S80 camera was employed to take images of tea leaves in the field. While taking the images, we adopted the close-range mode, turned off the flashlight to eliminate the impact of the camera’s light on the color of tea leaves. Moreover, the pictures should be taken in diffuse natural light without direct sunlight and with a focus distance of 15 cm and a resolution of 2592 × 1944, and the result of the segmentation is shown in Figure 4.

In the same conditions of imaging, the machine can automatically select the seed region for growth, but when the difference in lighting conditions is too significant or the tea is in different varieties, manual operation should be involved in the first round of seed selection.

### 5.2. Extraction of the Feature Parameters

The key technique in three-dimensional modeling of tea shoots is to extract the tea shoot parameters and construct a mathematical model to describe the tea-shoot shapes and changes, thereby drawing the realistic three-dimensional form of tea shoots in computer with the help of the tea-leaf images [25,26]. During the extraction of the feature parameters, binarization processing is applied to the tea-shoot images acquired after segmentation, the bud shape and branch contours of the tea shoots are acquired using the boundary following algorithm [27], and then the vein and branch axis are worked out by the central axis algorithm [28]. Then, by use of the bud and branch skeleton map, the leaf width, leaf length, branch length and average diameter are calculated, and the leaf angle is figured out according to the vein and branch axis. Finally, the acquired parameter data is matched with the data regarding leaves and branches in the database.

The contour of tea leaves acquired in the images is formed by a series of pixels, and thus it can be regarded as a sequence of one-pixel. Curve subdivision is used to reduce the number of tops and line segments. The curve subdivision algorithm keeps correcting the initial point set and makes them close to a smooth curve, thereby forming an accurate approximation consisting of a good many pixel points. Also, it was corrected according to the veins to ensure the consistency between it and the true leaf structure.

During the construction of the tea-leaf model database, the tea leaves were laid on a white background and photographed from different angles, and then the model database of tea leaves was acquired after removing the background. After segmentation, the tangent at the bottom of vein was made perpendicular to the long axis of the leaf, thereby being convenient for the tea-leaf shape recovery in three-dimensional modeling. In the experiment, the acquired parameter is in pixel units, and Table 1. shows the main parameter values in this experiment.

### 5.3. The Three-Dimensional Modeling of Tea-Shoot Shapes

In appearance, a tea-shoot branch is just a short segment and looks like a cylinder. For its modeling, we usually take the stretching algorithm. That is, the organs of cylindrical graphics can be formed if taking the section graphics as the tracks and giving the stretching height. We model each branch as a generalized cylinder, with the skeleton being a 3D spline curve. The cylindrical radius can be spatially varying. It is specified at each endpoint of each branch, and linearly interpolated between the endpoints.

Since leaves in the same plant are typically very similar, we adopt the strategy of extracting a generic leaf model from a sample leaf and using it to fit the same kind of leaves. In the three-dimensional Modeling, the best choice is to use the original leaf images and, in the three-dimensional simulation of growth, we chose the most similar leaf images that were acquired according to the growth model. For a tea leaf that can be regarded as a thin slice, the major characteristics are reflected on its surface, while the thickness of leaf can be ignored.

**Figure 6.**
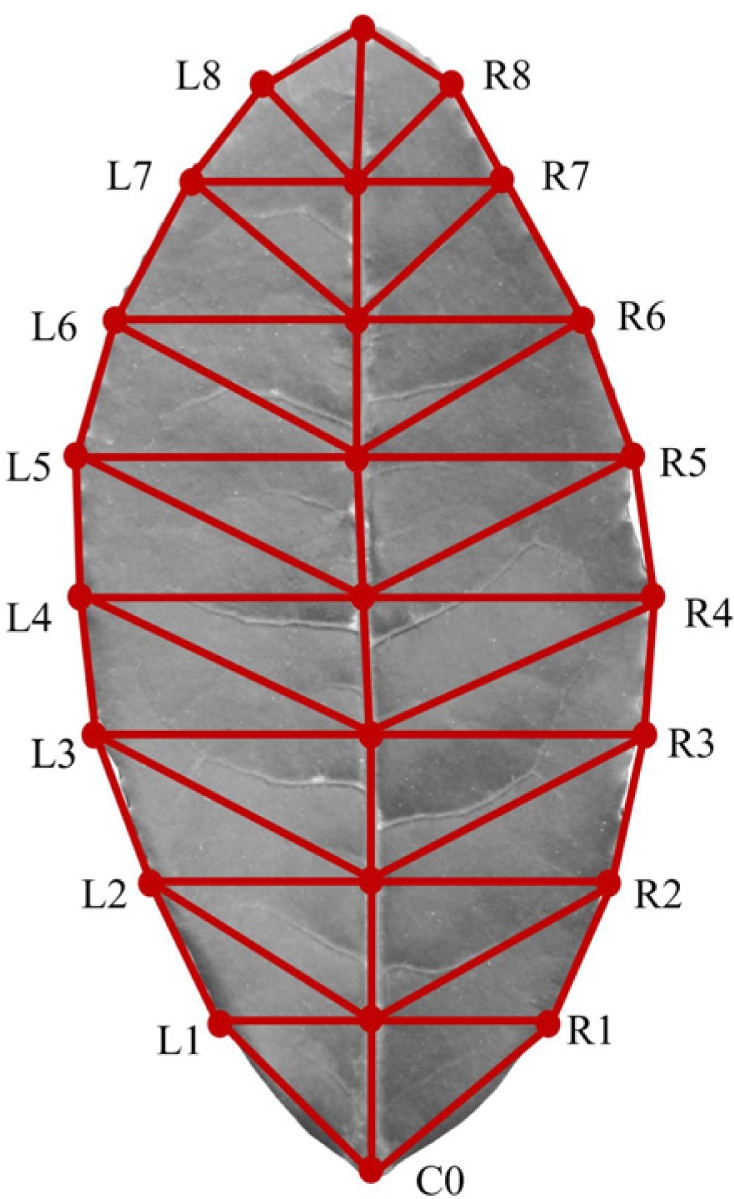
Matching schematic diagram of the tea leaf.

In the matching computation of tea leaves, the geometric shape of tea leaves, in short, consists of vein and edge outline, and the head and tail of a vein is connected with the edge outline. As shown in Figure 6, the tea leaf can be divided into several triangles by connecting the points on the vein and edge outline (there are 8–10 vertices along every edge).

The texture and boundary associated with the leaf are taken to be the model of the leaf. The model is automatically subdivided to increase the accuracy of the model, depending on the density of points in the group. The texture of each leaf first inherits that of the generic model in modeling. The texture from the image segmentation is subsequently used to overwrite the default texture.

**Figure 7.**
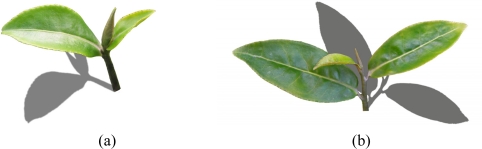
3D modeling image **(a)** and simulation image **(b)** of tea-shoot.

Once the branching structure is finalized, each leaf is automatically connected to the branch. The orientation of each leaf determined using extracted parameters. The tea-shoot model is produced by assembling all the reconstructed branches and leaves. The 3D modeling image and its subsequent simulation image of growth are shown in Figure 7.

During the three-dimensional modeling of tea-shoot images, the three-dimensional images are quite consistent with the original images due to the parameter matching and adoption of the segmented tea-shoot leaves, and the constructed branches. However, the tea leaves with bending and curling surface have certain impact on some parameters (e.g., the central axis, leaf angle, *etc*.) in modeling, resulting in some differences in the acquisition and matching of parameters.

In the modeling of three-dimensional growth of the tea shoots, the reconstruction was conducted by taking the law of time-related changes in tea shoots into account, which is based on the originally acquired parameters of the leaves and branches of the tea shoots. In this case, four parameters (leaf length, leaf width, branch diameter and leaf inclination angle) were selected, and the image of the leaf with the most similar width and length in the database was chosen and then, according to the branch diameter, the branches were constructed and given texture mapping. After that, by combining the leaves and branches according to the changes in leaf sequence and leaf inclination angle, the three-dimensional images regarding tea-shoot growth were constructed.

By the segmentation and three-dimensional modeling of tea shoots, the status of tea shoots can be observed more directly and, plus the three-dimensional growth simulation, the growth state and picking time can be grasped more accurately.

## 6. Conclusions

In this paper, a method for three-dimensional modeling of tea-shoot shapes with images and calculation models was introduced. With image verification technology, the method gives the tea-shoot model a better visual effect and controllability and, moreover, helps the model avoid complicated calculations and measurements and reduce the number of images. Also, with this method, a mathematical model to describe the tea-leaf shapes was established. Having the shape parameters with clear biological significance, the model can be associated perfectly with the agricultural model and achieve the three-dimensional visualization of the tea-leaf shape formation with less information, and it has a high fidelity. Also, to some extent, the modeling method in this study can be changed, and it can simulate the growth of tea shoots, which can provide new ideas and means for the selection of crops’ morphological characteristics and three-dimensional modeling. Actually the growth rate of the tea shoots is different in different seasons, so the growth model in this case is not quite accurate, hence more precise growth simulation will be studied in future.

## Figures and Tables

**Figure 5.**
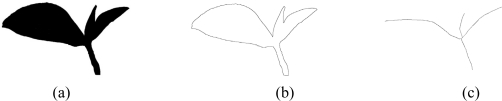
Tea-shoot images of binarization **(a)**; contour **(b)** and axis **(c)**.

**Table 1.** The main parameter values.

**Parameters**	**Value**

Leaf length of the first tender leaf	417
Leaf width of the first tender leaf	118
Leaf inclination angle of the first tender leaf	49.8°
Leaf length of the second tender leaf	779
Leaf width of the second tender leaf	325
Leaf inclination angle of the second tender leaf	131.1°
Branch length	321
Branch average diameter	51
Bud length	298
Bud average diameter	55
Bud inclination angle	75.8°
